# Supplementary figures and images for: Role of Endothelin-1 in Right Atrial Arrhythmogenesis in Rabbits with Monocrotaline-Induced Pulmonary Arterial Hypertension
Source: Int J Mol Sci. 2022 Sep 20;23(19):10993. doi: 10.3390/ijms231910993 (PMC9569916; doi:10.3390/ijms231910993)

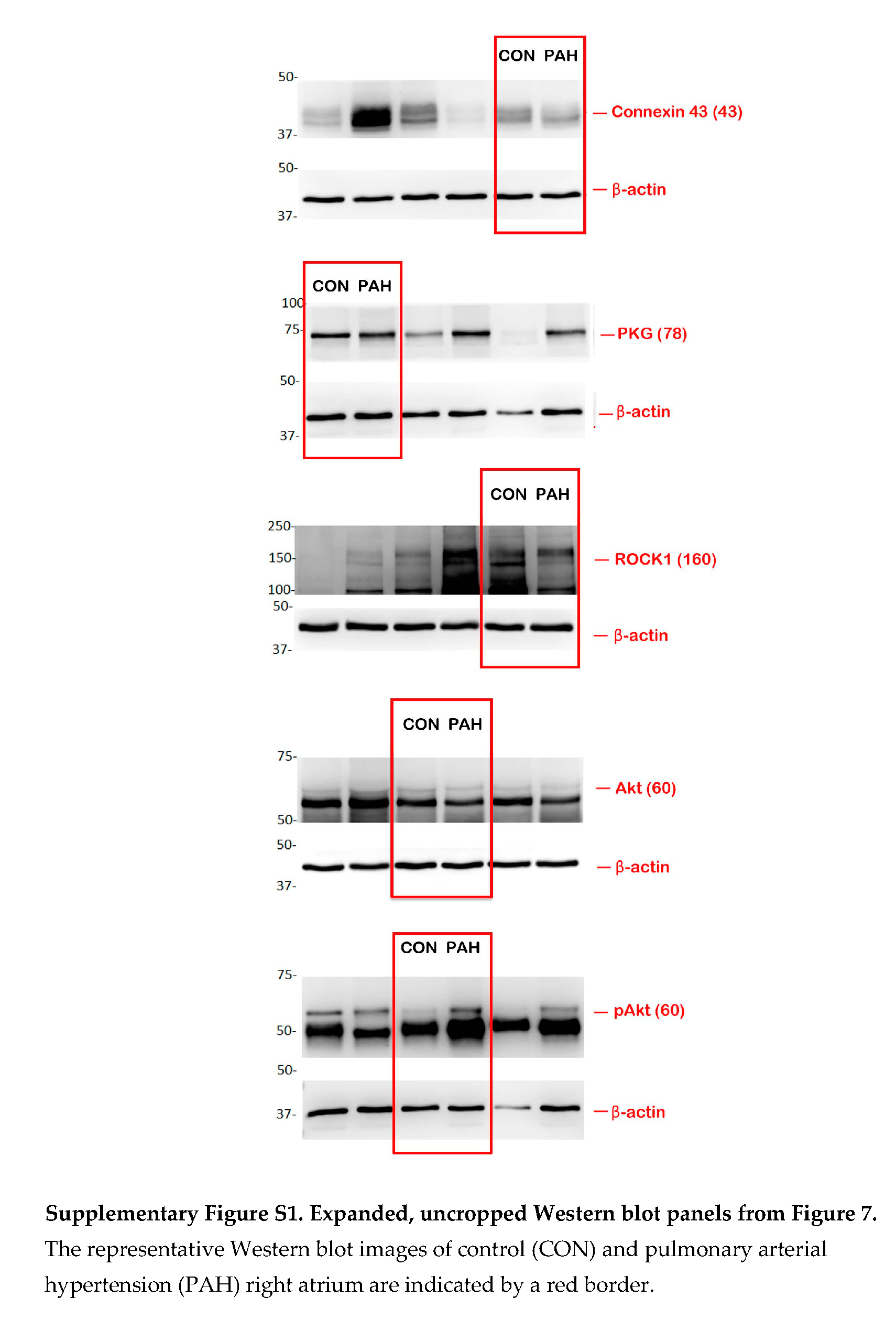

Supplement: Supplementary file 1 [file ijms-23-10993-s001.zip › ijms-1876785-supplementary.tif]
